# Salvinorin A Inhibits Airway Hyperreactivity Induced by Ovalbumin Sensitization

**DOI:** 10.3389/fphar.2016.00525

**Published:** 2017-01-13

**Authors:** Antonietta Rossi, Elisabetta Caiazzo, Rossella Bilancia, Maria A. Riemma, Ester Pagano, Carla Cicala, Armando Ialenti, Jordan K. Zjawiony, Angelo A. Izzo, Raffaele Capasso, Fiorentina Roviezzo

**Affiliations:** ^1^Department of Pharmacy, University of Naples Federico IINaples, Italy; ^2^Department of BioMolecular Sciences, Division of Pharmacognosy and the Research Institute of Pharmaceutical Sciences, School of Pharmacy, University of MississippiUniversity, MS, USA; ^3^Department of Agricultural Sciences, University of Naples Federico IIPortici, Italy

**Keywords:** Salvinorin A, leukotrienes, asthma, airway hyperreactivity, mast cells

## Abstract

Salvinorin A, a neoclerodane diterpene isolated from *Salvia divinorum*, exerts a number of pharmacological actions which are not solely limited to the central nervous system. Recently it has been demonstrated that Salvinorin A inhibits acute inflammatory response affecting leukotriene (LT) production. Since LTs are potent lipid mediators implicated in allergic diseases, we evaluated the effect of Salvinorin A on allergic inflammation and on airways following sensitization in the mouse. Mice were sensitized with s.c. injection of ovalbumin (OVA) on days 1 and 8. Sensitized mice received on days 9 and 12 on the shaved dorsal surface air administration to induce the development of the air-pouches. On day 15 animals were challenged by injection of OVA into the air-pouch. Salvinorin A, administered (10 mg/kg) before each allergen exposure, significantly reduced OVA-induced LT increase in the air pouch. This effect was coupled to a reduction in cell recruitment and Th2 cytokine production. In another set of experiments, mice were sensitized with OVA and both bronchial reactivity and pulmonary inflammation were assessed. Salvinorin A abrogated bronchial hyperreactivity and interleukin (IL)-13 production, without effect on pulmonary inflammation. Indeed cell infiltration and peribronchial edema were still present following diterpenoid treatment. Similarly, pulmonary IL-4 and plasmatic IgE levels were not modulated. Conversely, Salvinorin A significantly reduced LTC_4_ production in the lung of sensitized mice. Finally mast cell activity was evaluated by means of toluidine blue staining. Data obtained evidenced that Salvinorin A significantly inhibited mast cell degranulation in the lung. Our study demonstrates that Salvinorin A inhibits airway hyperreactivity induced by sensitization by inhibition of LT production and mast cell degranulation. In conclusion Salvinorin A could represent a promising candidate for drug development in allergic diseases such as asthma.

## Introduction

The plant *Salvia divinorum*, that occurs naturally in Mexico, has been used for centuries to facilitate spiritual experiences in religious rituals as well as employed by shamans for the cure of various disorders, including those characterized by having an inflammatory/allergic component (Vortherms and Roth, 2006; Mahendran et al., 2016). *S. divinorum* is actually primarily used by adolescent and young adults for its hallucinogenic properties with a prevalence of use ranging, for example, from 1.3% among adults in the USA to 11% of attendees to rave musical events in Italy (Mahendran et al., 2016). The main active ingredient of the plant is the neoclerodane diterpenoid Salvinorin A. Pharmacodynamic studies have shown that Salvinorin A is a potent and selective kappa opioid receptor agonist (Roth et al., 2002; Chavkin et al., 2004), may exert CB1-like effects, without being able to activate such receptors (Braida et al., 2007; Capasso et al., 2008; Fichna et al., 2012; Guida et al., 2012). In addition Salvinorin A has demonstrated to exert anti-inflammatory actions (Aviello et al., 2011; Rossi et al., 2016). Recently, we have demonstrated that its anti-inflammatory properties, at least in part, were related to ability to inhibit leukotriene (LT) biosynthesis (Rossi et al., 2016).

Leukotrienes are crucial mediators of allergic diseases, such as bronchial asthma, allergic rhinitis, and urticaria (Chen et al., 1994; Schauberger et al., 2016). They are synthesized from arachidonic acid predominantly by eosinophils, mast cells and macrophages in response to a variety of stimuli. Five-lipoxygenase, enzyme responsible of LT biosynthesis, converts the fatty acid into LTA_4_, the common precursor for LTB_4_ and LTC_4_. Subsequently, LTC_4_ is converted to LTD_4_ and LTE_4_ (cysteinyl-LTs; cys-LTs). They induce bronchoconstriction, inflammatory cell recruitment and plasma extravasation, and drive tissue edema, all these are classical signs of allergic pulmonary inflammation (Thivierge et al., 2001; Singh et al., 2013; Schauberger et al., 2016). Furthermore, following allergen exposure mast cells generate large amounts of cys-LTs, which in turn induce an autocrine-type amplification of Th2 response (Vargaftig and Singer, 2003; Kim et al., 2006). In fact activated mast cells produce several cytokines among which are IL-4 and -13, which are crucial for the development of asthma features. In particular there is evidence that IL-4 is crucial for Th2-cell differentiation from naive T cells. In addition IL-4 causes isotype class-switching of B cells toward IgE synthesis and it is involved in mast-cell recruitment and airway hyperresponsivity (Herz et al., 1998; Ryzhov et al., 2004; Chung, 2015; Ul-Haq et al., 2016). However, on the other hand, there is evidence that IL-4 may also reduce the activation of memory CD8 T cells and their following differentiation in NK cells, affecting in this way the immune response to pathogens. This could also explain the correlation, based also on clinical evidence, between a strong Th2 immune response (characterized by high IL-4 levels) and chronic parasitic infections (Actor et al., 1993; Ventre et al., 2012), indeed high levels of IL-4 may deviate the host respone toward a Th2 type. On the other hand, low levels of IL-4 may increase the susceptibility to autoimmune diseases (Hill and Sarvetnick, 2002; Ventre et al., 2012).

Because LTs play a fundamental role in the pathogenesis of asthma and other allergic diseases (Chen et al., 1994; Schauberger et al., 2016), in the present study, we have investigated the effect of Salvinorin A on airways following OVA sensitization. Possibly relevant to our study, it is intriguing the observation that Internet sites report recipes for home-made preparations of *S. divinorum* advocated to cure bronchial asthma.

Salvinorin A reduced bronchial hyperreactivity by inhibition of pulmonary mast cell degranulation and in turn, of IL-13 and LTC_4_ production.

## Materials and Methods

### Materials

Salvinorin A was isolated from leaves of *S. divinorum*, extracted and purified (purity: 99% by HPLC) as described in detail elsewhere (Capasso et al., 2006). All other reagents and fine chemicals were obtained from Sigma-Aldrich (Milan, Italy).

### Animals

Female BALB/c mice (18 ± 2 g body weight, Charles River, Calco, Italy) were housed in a controlled environment (21 ± 2°C) and provided with standard rodent chow and water. All animals were allowed to acclimate for 4 days prior to experiments and were subjected to 12 h light – 12 h dark schedule. Experiments were conducted during the light phase. The experimental procedures, according to Italian (DL 26/2014) and European (n. 63/2010/UE) regulations on the protection of animals used for experimental and other scientific purposes, were approved by the Italian Ministry.

### Sensitization and Drug Treatment

**FIGURE 1:**
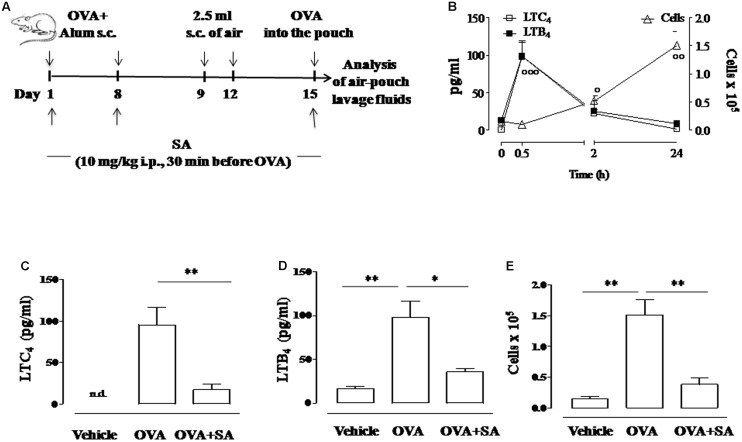
**Effect of Salvinorin A on LT production and cell recruitment in allergen-induced inflammation in air pouch. (A)** Scheme of air pouch model. Animals were injected with 0.4 ml s.c. of a suspension containing 100 μg of OVA absorbed to 3.3 mg of aluminium hydroxide gel on days 1 and 8. Then they received at days 9 and 12 on the shaved dorsal surface 2.5 ml s.c. of air. On day 15 animals were challenged by injection into the air-pouch with 0.4 ml of sterile saline alone (vehicle) or containing 10 μg OVA. Salvinorin A (OVA+SA; 10 mg/kg) or vehicle (dimethyl sulfoxide 4%, 0.5 ml; OVA) were administered i.p. 30 min before each OVA administration. **(B)** Time-course of LTC_4_ and LTB_4_ levels and of cell recruitment in the lavage fluid of air pouch following OVA challenge. **(C)** LTC_4_, and **(D)** LTB_4_ levels were quantified in lavage fluid of air pouch 30 min after OVA challenge, while **(E)** cell recruitment was quantified 24 h after OVA challenge. Data are expressed as means ± SEM from *n* = 6 animals for each group. °*p* < 0.05; ^∘∘^*p* < 0.01; ^∘∘∘^*p* < 0.001 vs. time 0 h; ^∗∗^*p* < 0.01; ^∗^*p* < 0.05.

Animals were injected with 0.4 ml s.c. of a suspension containing 100 μg of OVA absorbed to 3.3 mg of aluminium hydroxide gel on days 1 and 8 (OVA-sensitized mice) (**Figure 1A**) (Das et al., 1997; Roviezzo et al., 2007, 2015; Sullo et al., 2013). Salvinorin A (10 mg/kg; Rossi et al., 2016) or vehicle (dimethyl sulfoxide 4%, 0.5 ml) were administered i.p. 30 min before each OVA administration.

### Air Pouch Model

Mice, sensitized as described above, received on days 9 and 12 on the shaved dorsal surface, 2.5 ml s.c. of air to initiate the development of the air-pouches as described previously (Das et al., 1997) (**Figure 1A**). On day 15 (6 days after the first air injection) animals were challenged by injection into the air-pouch with 0.4 ml of sterile saline alone or containing 10 μg OVA. At different time-points (30 min, 2 or 24 h) after OVA or saline injection into the air-pouch, mice were sacrificed by exposition to CO_2_. Air-pouches were washed with 1 ml phosphate-buffered saline (pH = 7.4). Lavage fluids were centrifuged at 300 × *g* for 10 min at 4°C. Supernatants were then collected and stored at -80°C until assayed for LTs (Cayman Chemical; BertinPharma, Montigny Le Bretonneux, France), IL-4 and IL-13 evaluation by ELISA kits according to manufacturer’s instructions. Levels were expressed as pg/ml. Cell pellets were resuspended in phosphate-buffered saline and total cell counts were performed following Trypan blue staining.

### Bronchial Reactivity

Ovalbumin-sensitized mice were sacrificed on day 15 and 22 by cervical dislocation, exsanguinated, and lungs were removed. Main bronchi (22 days after sensitization) were rapidly dissected and cleaned from fat and connective tissue. Rings of 1–2 mm length were cut and mounted in 2.5 ml isolated organ baths containing Krebs solution, at 37°C, oxygenated (95% O2 and 5% CO_2_), and connected to an isometric force transducer (type 7006, Ugo Basile, Comerio, Italy) associated to a Powerlab 800 (AD Instruments). Rings were initially stretched until a resting tension of 0.5 g was reached and allowed to equilibrate for at least 30 min during which tension was adjusted, when necessary, to a 0.5 g and bathing solution was periodically changed. In each experiment bronchial rings were previously challenged with acetylcholine (10^-6^ mol/L) until a reproducible response was obtained. Subsequently, after tissue washing, a cumulative concentration response curve to carbachol (10^-9^ – 3 × 10^-6^ M) was performed. Results were expressed as dine *per* mg tissue.

### IgE, Cytokine, and LT Measurements

In another set of experiments OVA-sensitized mice were sacrificed at 15 days to take pulmonary tissues and blood for biochemical studies and IgE evaluation, respectively. Plasma IgE levels were measured by means of ELISA using matched antibody pairs (BD Pharmingen, Franklin Lakes, NJ, USA). Each lung was divided into two parts. One part was frozen in liquid nitrogen for 2 h before storage at -80°C and subsequently homogenate for cytokine and LT measurements by ELISA, and the other was fixed in 10% neutralized buffered formalin for histopathological evaluation. Levels of LTC_4_ and cytokines were expressed as pg/mg of tissue.

### Lung Histology

Lung sections were cut (7 μm thick) and stained with H&E for morphological analysis. Mast cell degranulation was evaluated following the method described by Iuvone et al. (1999). In brief, it was calculated the percentage of light blue stained cells following toluidine staining, i.e., degranulated mast cells, on the total number of mast cells, per mm^2^. Non-degranulated mast cells appeared deep blue stained. The sections were analyzed by blinded operators using a standard light microscope (20× magnification, for H&E staining, and 40× magnification, for toluidine blue staining) and photographed under low power. Images were taken by a Leica DFC320 video-camera (Leica, Milan, Italy) connected to a Leica DM RB microscope using the Leica Application Suite software V.4.1.0.

### Myeloperoxidase Activity

Myeloperoxidase activity in lung tissues harvested 15 days after OVA sensitization was determined as previously described (Rossi et al., 2016). Each piece of tissue was weighed and then homogenized in a solution containing 0.5% hexadecyltrimethylammonium bromide dissolved in 10 mM phosphate-buffered saline (pH 7) and centrifuged (30 min at 20,000 × *g* at 4°C). An aliquot of the supernatant was then allowed to react with a solution of tetramethylbenzidine (1.6 mM) and 0.1 mM H_2_O_2_. The rate of change in absorbance was measured spectrophotometrically at 650 nm. MPO activity was defined as the quantity of enzyme degrading 1 μmol of peroxide per minute at 37°C and was expressed in units *per* gram weight of wet tissue.

### Statistical Analysis

Data are expressed as mean ± standard error of the mean (S.E.M.) of *n* observations, were *n* represents the number of animals (at least of six per group for each data set). Statistical analysis has been performed by using *t*-test or two-way analysis of variance (ANOVA) for multiple comparisons followed by Bonferroni’s post-test [GraphPad Prism 5.0 software (San Diego, CA, USA)]. *Post hoc* tests were performed when ANOVAs indicated that a significant difference existed between groups. All statistical tests performed showed no significant variance in data set homogeneity. Data were considered statistically significant when a value of at least *p* < 0.05 was achieved.

## Results

### Salvinorin A Inhibits Allergen-Induced LT Production in Air Pouch Model

Previously, we demonstrated that Salvinorin A reduced LT synthesis in experimental models of acute inflammation (Rossi et al., 2016). Therefore, we investigated the possibility that this natural diterpene might affect LT production also during allergic inflammation. For this purpose, we used the air pouch model in OVA-sensitized and challenged mice (Das et al., 1997) (**Figure 1A**). We chose this model since air pouch provides a convenient cavity from which mediators and cells can be easily harvested. OVA administration in the dorsal air pouch of sensitized-mice caused a significant increase in LTC_4_ and LTB_4_ production, which peaked at 30 min and returned to basal levels after 24 h (**Figure 1B**). Mouse pre-treatment with Salvinorin A (10 mg/kg, i.p., 30 min prior each OVA injection) nearly reduced LTC_4_ and LTB_4_ production in the lavage fluids of air pouch (**Figures 1C,D**).

### Salvinorin A Blunts Allergen-Induced Increase in Cell Recruitment in Air Pouch Model

In order to assess if the inhibition of LT production was associated to a reduced inflammatory response, we evaluated also cell recruitment. Injection of OVA in the air pouch provoked an intense allergen-dependent cell accumulation that was significative 2 h after challenge, showing a peak at 24 h (**Figure 1B**). Salvinorin A (10 mg/kg, i.p.; 30 min prior each OVA injection) significantly inhibited OVA-induced cell infiltration in the air pouch (**Figure 1E**).

### Salvinorin A Abolishes Allergen-Induced Cytokine Production in Air Pouch Model

**FIGURE 2:**
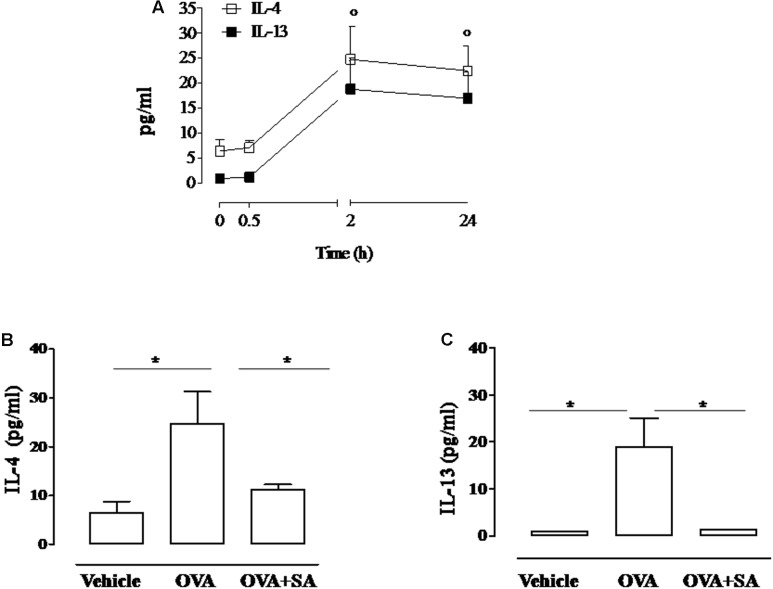
**Effect of Salvinorin A on IL-4 and IL-13 in allergen-induced inflammation in air pouch.** Salvinorin A (OVA+SA; 10 mg/kg) or vehicle (dimethyl sulfoxide 4%, 0.5 ml; OVA) were administered i.p. 30 min before each OVA administration. **(A)** Time-course of IL-4 and IL-13 in the lavage fluid of air pouch after OVA challenge. **(B)** IL-4 and **(C)** IL-13 were quantified in lavage fluid of air pouch 2 h after OVA challenge by ELISA. Data are expressed as means ± SEM from *n* = 6 animals for each group. °*p* < 0.05 vs. time 0 h; ^∗^*p* < 0.05.

Since allergen-induced cell recruitment is sustained by Th2 cytokines, we evaluated if Salvinorin A affected the production of these mediators. Two hours following OVA challenge, we observed a significant increase of IL-4 and IL-13 levels into the pouch of sensitized mice (**Figure 2A**). Salvinorin A (10 mg/kg, i.p.; 30 min prior each OVA injection) administration abolished OVA-induced increase of both Th2 cytokines (**Figures 2B,C**).

### Salvinorin A Counteracts Bronchial Hyperreactivity in Sensitized Mice

**FIGURE 3:**
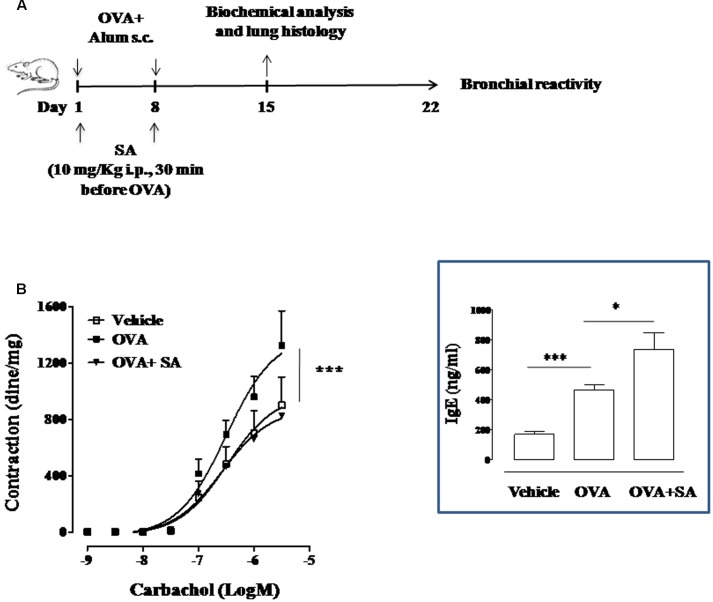
**Effect of Salvinorin A on allergen-induced bronchial hyperreactivity. (A)** Scheme of sensitization and drug treatment. Mice were injected with 0.4 ml s.c. of a suspension containing 100 μg of OVA absorbed to 3.3 mg of aluminium hydroxide gel on days 1 and 8. After 15 and 22 days after OVA sensitization mice were sacrificed. Salvinorin A (OVA+SA; 10 mg/kg) or vehicle (dimethyl sulfoxide 4%, 0.5 ml; OVA) were administered i.p. 30 min before each OVA administration. **(B)** Bronchial reactivity to carbachol were evaluated 22 days after OVA injection. Insert to figure shows IgE levels in the plasma. Data are expressed as means ± SEM from *n* = 6 animals for each group. ^∗^*p* < 0.05; ^∗∗^*p* < 0.001.

Since Salvinorin A confirmed its ability to affect LT and IL-13 production in an allergic environment, we went on evaluating its effect on bronchial reactivity of OVA-sensitized mice. To this aim, we exposed mice to OVA and part of these were pre-treated with Salvinorin A (10 mg/kg, i.p.; 30 min prior each OVA injection) (**Figure 3A**). Bronchial reactivity to carbachol was assessed. We found that bronchi, excised from OVA-sensitized mice, showed a significant increased reactivity to carbachol (**Figure 3B**) compared to vehicle group. Salvinorin A pre-treatment reported bronchial reactivity to values measured in vehicle treated mice (**Figure 3B**). Conversely, Salvinorin A did not inhibit OVA-induced IgE up-regulation in the plasma, it rather further increased their levels (insert to **Figure 3**).

### Salvinorin A Does Not Affect Pulmonary Inflammation in Sensitized Mice

**FIGURE 4:**
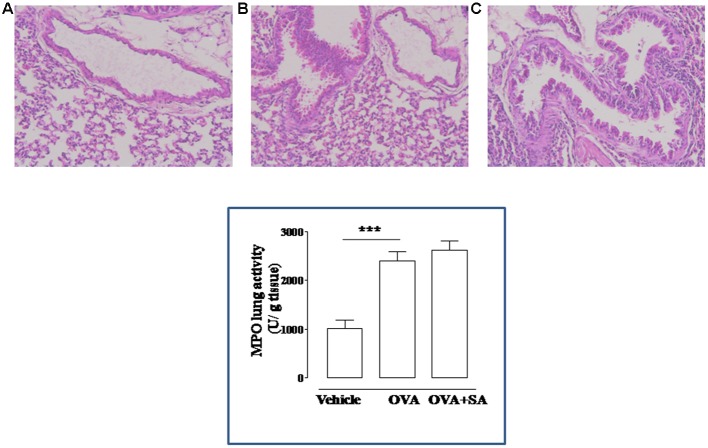
**Effect of Salvinorin A on allergen-induced pulmonary inflammation.** Salvinorin A (OVA+SA; 10 mg/kg) or vehicle (dimethyl sulfoxide 4%, 0.5 ml; OVA) were administered i.p. 30 min before each OVA administration. H&E staining of lung tissue harvested 15 days after OVA sensitization. H&E staining showing significant perivascular and peribronchial infiltrates of inflammatory cells in OVA-sensitized mice **(B)**. Vehicle group showed normal appearance of lung parenchyma **(A)**. Salvinorin A did not inhibit infiltration of inflammatory cells in the lung tissue **(C)**. Insert to figure shows the MPO activity in the lung tissues. ^∗∗∗^*p* < 0.001.

**FIGURE 5:**
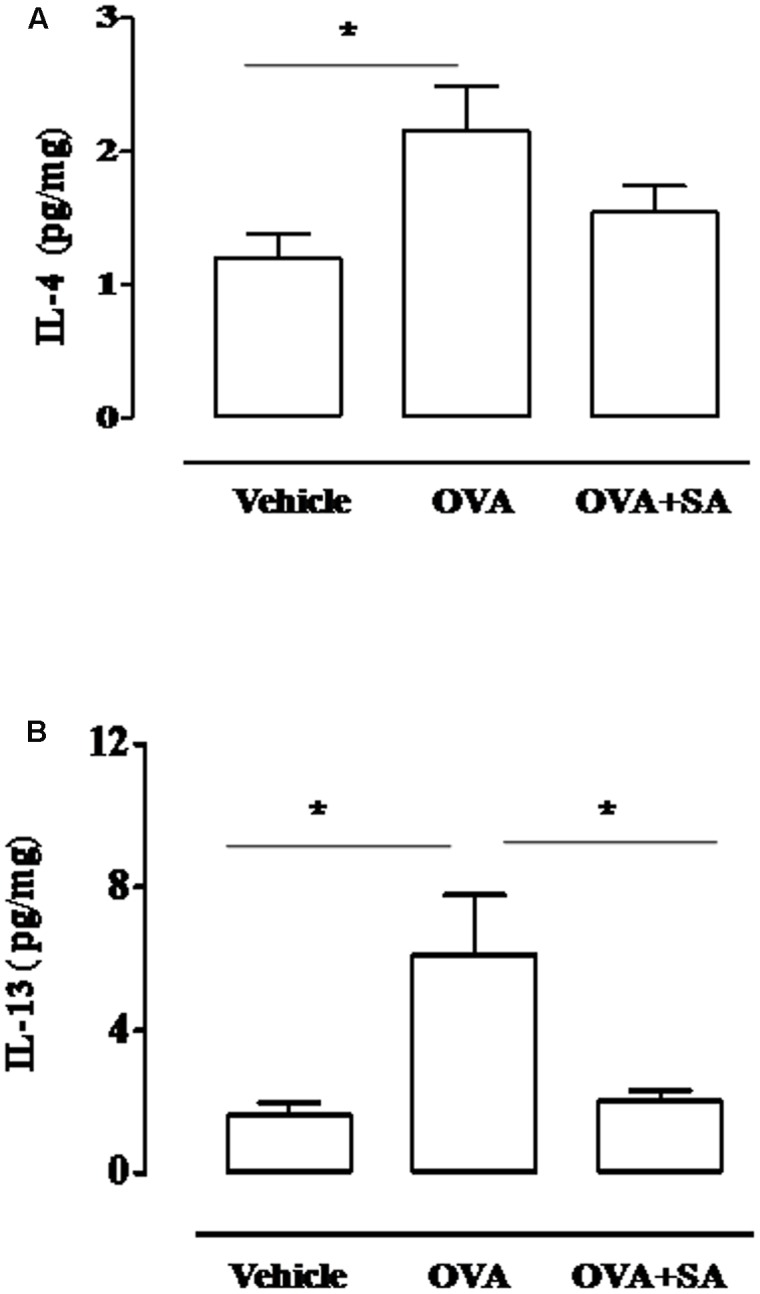
**Effect of Salvinorin A on IL-4 and IL-13 pulmonary levels in sensitized-mice.** Salvinorin A (OVA+SA; 10 mg/kg) or vehicle (dimethyl sulfoxide 4%, 0.5 ml; OVA) were administered i.p. 30 min before each OVA administration. **(A)** IL-4 and **(B)** IL-13 were quantified in lung tissue harvested 15 days after OVA sensitization by ELISA. Data are expressed as means ± SEM from *n* = 6 animals for each group. ^∗^*p* < 0.05.

Because bronchial hyperreactivity is one of the hallmarks of asthma and it is closely related to bronchial inflammation, we extend our study to the evaluation of pulmonary inflammation in sensitized mice. For this reason, sensitized mice were sacrificed at 15 days and pulmonary sections used for histology and biochemical analysis (**Figure 4**). The data obtained demonstrate that Salvinorin A (10 mg/kg, i.p.; 30 min prior each OVA injection) did not affect pulmonary inflammation, as highlighted by H&E staining (**Figure 4**). Extensive cell infiltration was still evident in lung sections of mice pre-treated with Salvinorin A (**Figure 4C**), in accordance with high MPO activity (insert to **Figure 4**). Furthermore, also peribronchial edema, present in OVA-sensitized mice (**Figure 4B**), persisted following Salvinorin A pre-treatment. In perfect tune with this data, pulmonary cytokine evaluation evidenced that Salvinorin A slightly, although not significantly, affected IL-4 up-regulation (**Figure 5A**). Conversely, Salvinorin A significantly reduced IL-13 lung levels accordingly to the inhibitory action on bronchial hyperreactivity (**Figure 5B**).

### Salvinorin A Inhibits Pulmonary Mast Cell Degranulation and LTC_4_ Levels in Sensitized Mice

**FIGURE 6:**
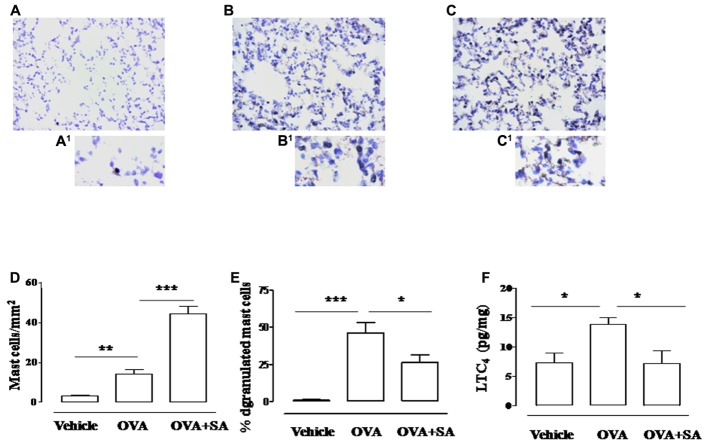
**Effect of Salvinorin A on pulmonary mast cell recruitment and degranulation.** Salvinorin A (OVA+SA; 10 mg/kg) or vehicle (dimethyl sulfoxide 4%, 0.5 ml; OVA) were administered i.p. 30 min before each OVA administration. **(B)** In lung of OVA sensitized mice (15 days) there was an increased mast cell recruitment and degranulation evaluated by Toluidine blue staining (light blue). Lung tissue harvested from Salvinorin A-treated mice **(C)** showed a further increase in mast cell recruitment but mostly mast cells were not degranulated (deep blue). **(A)** Control lung tissue. **A^1^–C^1^** are field magnification of **A**, **B** and **C**, respectively. **(D)** Quantification of cell recruitment and **(E)** percentage of mast cell degranulation evaluated as ratio between degranulated and total mast cells. **(F)** LTC_4_ levels were quantified in lung tissue by ELISA. Data are expressed as means ± SEM from *n* = 6 animals for each group. ^∗^*p* < 0.05; ^∗∗^*p* < 0.01; ^∗∗∗^*p* < 0.001.

Systemic exposure to allergens results in both the production of IgE against multiple antigen epitopes of several different antigens and the development of long-term changes in the involved tissues, including changes in mast cell number, tissue distribution (with mast cells in the epithelium and the smooth muscle layer) and phenotype. Binding of IgE to Fc𝜀RI on mast cells, which are normally located in airway tissues, upregulates Fc𝜀RI surface expression and sensitizes these cells to respond when later exposed to specific antigens, but also enhance cytokine production and survival (Bradding et al., 2006). Since direct correlation between mast cell infiltration and bronchial hyperreactivity exists, we focused on Salvinorin A effect on mast cell function. As highlighted by toluidine staining, OVA sensitization significantly increased mast cell recruitment (**Figures 6B,B^1^,D**) into the lung, as well as their degranulation (**Figures 6B,B^1^,E**) when compared with control (**Figures 6A,A^1^**). Salvinorin A (10 mg/kg, i.p.; 30 min prior each OVA injection) did not inhibit mast cell recruitment, but it further increased it (**Figures 6C,C^1^,D**). Conversely, Salvinorin A inhibited mast cell degranulation (**Figures 6C,C^1^,E**). Since LTC_4_ is recognized as the main mediator released by mast cells in allergic asthma, we measured its levels in the lung; as expected, sensitized mice showed an increased pulmonary levels of LTC_4_, effect that was abrogated when mice were pre-treated with Salvinorin A (**Figure 6F**).

## Discussion

Recent studies in animal models have revealed that Salvinorin A exerts a number of pharmacological actions of potential therapeutic interest which are not solely limited to the central nervous system (Butelman and Kreek, 2015). These include attenuation of inflammation (Aviello et al., 2011; Rossi et al., 2016), inhibition of intestinal motility (Capasso et al., 2008), and antipruritic effects (Salaga et al., 2015). The present study has further expanded the range of the pharmacological actions of this diterpenoid by evaluating its role in an allergic environment.

Our previous work has provided a deeper insight into the anti-inflammatory molecular mechanisms of Salvinorin A, by evidencing its ability to inhibit LT production and LT-related inflammatory parameters (Rossi et al., 2016). Starting from such evidence, and considering that LTs are key mediators of allergic inflammation and critical regulators in the development of asthma (Singh et al., 2013), we have investigated the effects of Salvinorin A on allergic inflammation and on airways following OVA-sensitization in the mouse.

First, we evaluated if the inhibitory effect of Salvinorin A on LT production persisted also during an inflammatory response induced by allergic challenge. Specifically, we have used a model of allergen-induced inflammation into mouse dorsal air pouch (Das et al., 1997). The air pouch provides a convenient cavity from which cells and inflammatory mediators can be easily harvested. It is important to mention that the cell population found in 6-day-old air pouches of sensitized mice consists essentially of mononuclear cells (Das et al., 1997). According to previously published data, injection of OVA into the pouch of sensitized-mice provoked a slight air pouch cellular influx starting from 2 h with a peak at 24 h after OVA challenge. Interestingly, the accumulation of cells appeared as a delayed event as compared to the rapid LT production (30 min after OVA challenge), suggesting that LT production by resident cells is a key event for the following cell recruitment. Indeed, pre-treatment of sensitized mice with Salvinorin A reduced both LT increase at the early time (30 min) and the following cell infiltration into air pouch (24 h).

It has been widely demonstrated in various animal models that allergic inflammation is primarily dependent on Th2 response, where LT play an important role. In fact the Th2 type inflammatory reaction, characterized by IL-4 and IL-13 production (Lloyd and Hessel, 2010), is suppressed in antigen-exposed mice deficient of LTC_4_ synthase (Kim et al., 2006). Besides it has been reported that LT modifier affect IL-4 and IL-13 production, but not other cytokines in an experimental model of allergic airway inflammation (Kawano et al., 2014). Accordingly, in our experimental conditions Salvinorin A significantly reduced IL-13 and IL-4 levels in the air pouch 2h following OVA administration.

Collectively, these results demonstrate that Salvinorin A displays a significant control also of the allergic inflammation and its beneficial effect is correlated to LT inhibition.

In order to further corroborate our hypothesis and to assess its potential application, we looked at the effects of Salvinorin A on airways following OVA sensitization. In particular, we used as experimental approach a systemic exposure of mice to OVA that induces an significant increase in plasmatic IgE level. This effect is coupled to a significant increase in bronchial hyperreactivity and pulmonary inflammation associated to pulmonary mast cell recruitment (Roviezzo et al., 2015). Measurements of bronchial reactivity *in vitro* evidenced a significant increase in carbachol-induced contractions, that was reversed by Salvinorin A. This effect was associated to a reduction of IL-13 levels in the lung of Salvinorin A pre-treated mice. Our finding is in agreement with evidence that IL-13 is a cytokine specifically involved in bronchial hyperreactivity, with a mechanism independent of cell accumulation or airway mucus production (Venkayya et al., 2002).

Indeed, the diterpenoid had no effects on inflammatory response in the lung. Cell infiltration was still evident in pulmonary sections of lungs harvested from mice pre-treated with Salvinorin A, such as peribronchial edema. In perfect tune with this data and accordingly to the high level of IgE found in sensitized mice pre-treated with Salvinorin A, we observed a slightly, but not significant inhibition of IL-4 increase in the lung. Thus, Salvinorin A seems do not affect sensitization mechanisms, but interferes with effector mechanisms responsible of regulation of bronchial tone.

Surprisingly, the lack of effect of Salvinorin A on the development of allergic pulmonary inflammation is not in line with its effects on air pouch model, in which both cell recruitment and increased cytokine levels, triggered by allergen challenge, were inhibited by diterpenoid. This discrepancy might be due mainly to the differences between the air pouch cavity and airways in terms of resident and recruited cells. In addition, it is plausible that Salvinorin A regulates cytokine production and cell recruitment during the acute phase, but its relative role may be altered during chronic phase of allergic inflammation such as in the lung harvested from sensitized mice (15 days after), when other cell populations come into play. Accordingly, we also published data demonstrating that Salvinorin A inhibited neutrophil infiltration as well as MPO activity in the lung harvested from mice after pleurisy induction (Rossi et al., 2016). Also in this case the protective effects of Salvinorin A occurs at early time (4 h) following inflammatory stimulus.

In order to further gain insight into the cellular mechanisms underlying the therapeutic effects of Salvinorin A on airway hyperreactivity, we went on evaluating the role of mast cells. Although the role of mast cells in experimental asthma models is still controversial, recently several researchers have demonstrated the role of innate immune cells in asthma development and in this context, mast cells seem to play an important role, especially in the process of sensitization to allergen (Deckers et al., 2013). Indeed, mast cell infiltration in the smooth muscle is correlated with responsiveness to cholinergic stimuli as, we have already demonstrated in our experimental setting (Roviezzo et al., 2015). In particular, mast cells, armed with specific IgE and residing in the mucosa, serve as airway sentinels, sensing and responding to inhaled antigens. In patients with asthma, the IgE-mediated activation of these cells following allergen exposure induces release of vasoactive and smooth muscle-constricting mediators, that trigger acute airflow obstruction, as well as the production of bioactive lipids, cytokines, and chemokines (Bradding and Arthur, 2016). However, systemic exposure to allergens results in both the production of IgE against multiple antigen epitopes of several different antigens and the development of long-term changes in the involved tissues, including changes in mast cell number, tissue distribution (with mast cells in the epithelium and the smooth muscle layer) and phenotype. Binding of IgE to Fc𝜀RI on mast cells, which are normally located in airway tissues, upregulates Fc𝜀RI surface expression and sensitizes these cells to respond when later exposed to specific antigens, but, in mast cells, some IgE molecules can also enhance cytokine production and cell recruitment (Bradding et al., 2006). All these events explain the development of bronchial hyperactivity following sensitization also in absence of an airway challenge. Our results show that Salvinorin A significantly inhibited mast cell degranulation in the lung of sensitized mice. This regulatory effect on mast cells was in according to the recent published experimental data demonstrating that Salvinorin A analogs attenuate compound 48/80-induced itch responses in mice through a KOR-mediated mechanism (Salaga et al., 2015). The inhibitory action on mast cells, in our experimental conditions, was confirmed by the significant reduction of lung LTC_4_ levels. Conversely Salvinorin A induced a significant increase of mast cell number in lungs when compared to OVA-sensitized mice as well as plasmatic IgE levels. This increase could reflect a reduction of IgE bound to Fc𝜀RI on mast cell surface, consistent with inhibition of mast cell degranulation. On the other hand the increase in pulmonary mast cell infiltration could represent a rebound effect due to the inhibition of mast cell activity, e.g., degranulation. However, further studies will be necessary to clarify the molecular mechanisms underlying modulatory action on mast cells and the beneficial actions of Salvinorin A in asthma management. In particular it will be interesting to assess Salvinorin A effects also after repetitive airway challenge in OVA-sensitized mice or by using other allergenic stimuli such as house dust mite.

In summary, Salvinorin A does not interfere with sensitization mechanisms but significantly inhibits airway hyperreactivity and this effect is sustained by inhibition of mast cell degranulation/LT production. These characteristics, in addition to good inhaled pharmacokinetic profile (Johnson et al., 2016), make Salvinorin A a suitable and promising candidate for drug development in LT-related allergic inflammatory diseases such as asthma.

## Author Contributions

AR, RC, and FR designed and performed the experiments, analyzed the data, and wrote the manuscript; EC and CC performed and analyzed lung histology experiments; MR, RB, and EP assisted in animal experiments and biochemical analysis; JZ carried out the isolation of Salvinorin A from *Salvia divinorum*; CC, ArI, and AnI revised the manuscript. All authors read and approved the final manuscript.

## Conflict of Interest Statement

The authors declare that the research was conducted in the absence of any commercial or financial relationships that could be construed as a potential conflict of interest.

## Abbreviations

Cys-LTs: cysteinyl-leukotrienes
H&E: haematoxylin and eosin
IL: interleukin
LTs: leukotrienes
MPO: myeloperoxidase
OVA: ovalbumin
